# Attachment Theory and Spirituality: Two Threads Converging in Palliative Care?

**DOI:** 10.1155/2013/740291

**Published:** 2013-11-11

**Authors:** Cécile Loetz, Jakob Müller, Eckhard Frick, Yvonne Petersen, Niels Christian Hvidt, Christine Mauer

**Affiliations:** ^1^Ludwig Maximilians University of Munich, Clinic and Policlinic for Palliative Medicine, Germany; ^2^Munich School of Philosophy, Germany; ^3^Ludwig Maximilians University of Munich, Clinic and Policlinic for Palliative Medicine, Munich School of Philosophy, Germany; ^4^Hospital of the “Barmherzige Brüder,” Palliative Unit, Germany; ^5^Research Unit of Health, Man and Society, Institute of Public Health, SDU, Denmark; ^6^Freiburg Institute for Advanced Studies, Germany

## Abstract

The aim of this paper is to discuss and explore the interrelation between two concepts, attachment theory and the concept of spirituality, which are important to palliative care and to founding a multivariate understanding of the patient's needs and challenges. Both concepts have been treated by research in diverse and multiform ways, but little effort has yet been made to integrate them into one theoretical framework in reference to the palliative context. In this paper, we begin an attempt to close this scientific gap theoretically. Following the lines of thought in this paper, we assume that spirituality can be conceptualized as an adequate response of a person's attachment pattern to the peculiarity of the palliative situation. Spirituality can be seen both as a recourse to securely based relationships and as an attempt to explore the ultimate unknown, the mystery of one's own death. Thus, spirituality in the palliative context corresponds to the task of attachment behavior: to transcend symbiosis while continuing bonds and thus to explore the unknown environment independently and without fear. Spiritual activity is interpreted as a human attachment behavior option that receives special quality and importance in the terminal stage of life. Implications for clinical practice and research are discussed in the final section of the paper.

## 1. Introduction

The goal of palliative care is to improve the quality of life of terminally ill patients by preventing or relieving them from suffering. Relieving suffering does not only mean to insulate patients from physical pain. There are many more aspects which contribute to the patient's wellbeing such as social, psychological, and spiritual support [1]. This is why palliative medicine has to take a multidisciplinary approach to patient care. In the present paper, two important concepts come into focus, both founding a multivariate understanding of the patient's needs and challenges in the palliative situation: attachment theory and the concept of spirituality. Attachment Theory is a concept that is concerned with human relationship behavior in situations of loss, separation, or helplessness and can help to understand the patient's behavior, needs, and challenges [2, 3]. The patient's spirituality in health care and research is addressed by the field of Spiritual care. spiritual care is an interdisciplinary and cross-cultural discipline [4] in health care that scientifically addresses the spiritual and religious needs of patients and has become a part of medical care and education. Both concepts have been treated by palliative research in diverse the and multiform ways, as we will propound in the overview of research literature in the following section. But no effort has been made to integrate them into one theoretical framework in reference to the palliative context. The aim of this paper is to discuss the relevance of both concepts to palliative care, in order to develop a theoretical foundation for both aspects of the palliative situation concerned by Attachment Theory and spiritual care: the dynamics of human relationships and the dimension of spirituality when approaching the end of life. First, we will give an overview of the research literature on Attachment Theory and Spiritual Care in Palliative Care. Following this, we will discuss the relevance of Attachment Theory to Palliative Care. Then, we will discuss the role of spirituality in the palliative context with respect to the role of human and spiritual relationships. Finally, we will bring into dialogue the concepts of attachment and spirituality in order to emphasize their correspondence and mutual enrichment. We will interpret spirituality as an option of human attachment behavior that receives special importance in the terminal stage of life. For this reason, we will point out consequences for theoretical and practical treatment of spirituality in Palliative Care. 

## 2. Attachment Theory and Spiritual Care in the Palliative Context: An Overview of the Research Literature

### 2.1. Attachment Theory

While Attachment Theory is a very prominent concept in psychology, psychiatry, and psychotherapy [5–7], it has rarely been considered by research in Palliative Care, notwithstanding the fact that close relationships in Palliative Care are fundamentally important. Attachment patterns have an impact on illness behavior [8], on the physician-patient relationship [9], and thus on patient's communication and compliance. In the palliative situation, some attempts have been made to address family relationships and to facilitate communication [10, 11] or to identify determinants of depression and hopelessness in metastatic cancer patients [12] from an Attachment Theory perspective. Tan et al. [13] and Petersen and Koehler [2] made the effort to apply Attachment Theory to analyze interpersonal processes in Palliative Care and identified fundamental attachment patterns in patient's behavior. Milberg et al. [3] pointed out the importance of a “secure base” from a patient's and relatives' perspectives in palliative home care (a concept derived from Attachment Theory, as we shall see later). Due to the fact that the relevance of Attachment Theory to palliative research is an underrepresented issue, we will review some basic implications of the theory in this paper in order to integrate them with the concept of spirituality. 

### 2.2. Spiritual Care

There has been growing interest in spiritual care since the 1990s [14], and thus the spiritual needs of palliative patients and their relatives have become a significant concern in clinical practice and research. Different surveys demonstrate the need for religious and/or spiritual support by a sizable proportion of the palliative patient population. For example, Balboni et al. [15] reported that 86% of the cancer patients judged Spiritual Care as being an important part of medical care. Studies have shown that, for many patients, their spirituality is an important resource in coping with chronic disease [16–19]. This is since a patient's spiritual relations can facilitate treatment-related decision making and improve their compliance with medical care [20, 21], as well as offer consolation and confidence and thus significantly enhance quality of life in the terminal stage [22–25]. These findings are not only applicable to countries and regions with a strong religious tradition, but also to secular societies [26–28]. Spirituality concerns the religious as well as the nonreligious dimensions of human personality and is not reducible to specific religious dogma (for the difference between religion and spirituality see Hvidt et al. [29]). In this respect, Spiritual Care provides the opportunity for a cross-cultural and interreligious approach to Palliative Care [30]. When suffering from spiritual distress, patients tend to show increased physical and psychological symptoms, leading to an increased need for medical care services [31]. Thus, providing Spiritual Care can significantly reduce medical care costs in palliative medicine [32]. But what is the content of Spiritual Care? What are the spiritual themes, needs, and distresses of patients and how should they be addressed in research and clinical practice? Several approaches have been made to conceptualize spirituality in palliative care and will be reviewed later in this paper. 

### 2.3. Attachment Theory and Spirituality

In the concept of “Attachment to God,” Kirkpatrick and Shaver have made an effort to integrate the concepts of Attachment Theory and Spirituality [33, 34], although with no special attention to Palliative Care. Several studies found a correlation between a stable relationship to God and a secure attachment pattern [35–37]. Following this line of thought for a spiritual/religious person, a transcendent figure like God can adopt the role of an attachment figure, providing a secure base for personal development and compensating for dysfunctions in social relationships [38]. Nevertheless, little is known about this concept in the context of Palliative Care. An application of the concept of “Attachment to God” to Palliative Care surely would be fertile. In the palliative situation, attachment to a transcendent figure may be an important resource for patients in coping with their extraordinary situation and an important factor in providing a dignified parting. However, as we will discuss later, the concept of Spirituality can be understood more broadly than meaning a personal relationship to God or a higher being.

## 3. Attachment Theory and Palliative Care

### 3.1. Why Attachment Theory Is Relevant to Palliative Care

Moving toward the end of life, palliative patients are confronted with a deep intrusion into their structure of relationships. Associated persons will change their behavior. Relatives, friends, or colleagues will pay them special attention, possibly visiting them to spend some time, say good-bye, or pretend that there is nothing wrong. Perhaps some of them in their embarrassment will avoid any contact at all; some will become emotional or be accompanied by a melancholic graveness. Of course, some of the patient's associated persons will do well at dealing with the situation and will provide important support, while others will be a burden making unreasonable demands or being unable to empathize with the patient's situation. Final affairs will be put in order, but old conflicts can erupt as well. In whatever way the patient's personal environment adjusts to the situation, it happens with regard to the final separation that is imminent. For the patients, the palliative situation means an ineluctable abandonment of attachment to family and friends, a situation of loss. On top of which come the changes in the context of clinical care. When patients enter a palliative care ward, they are facing a medical environment different from that before. It is no longer about curing disease, but caring for the dying. Thus, the role of the doctor changes: he or she is no longer the lifesaver but a companion to someone who is dying, and this change may be accompanied by the patient's feelings of disappointment or abandonment.

With all these considerations in mind, it is crucial to take into account a concept that deals with human behavior in the situation of loss, separation, and helplessness, in order to understand the patient's behavior, needs, and challenges. This is why Attachment Theory is relevant to Palliative Care. 

### 3.2. What Is Attachment Theory?

Attachment Theory—initially developed by the British Psychoanalyst John Bowlby—describes the dynamics of relationships between individuals. According to this theory, attachment is defined as a fundamental, congenital need of human beings [39]. Developing a bond to a primary caregiver—mostly to the parents—in early infancy is crucial to laying the foundation for normal emotional, social, and cognitive development. Deprivation of such an Emotional bond to a consistent caregiver can result in so-called hospitalism, also known as adjustment disorder [40], which includes symptoms such as retarded physical and cognitive development and emotional and social disturbances, Based on the confidence children can acquire from parental care, they form a stable, internal representation or “working model” of interpersonal relationships with caregivers [13] which will later in life guide their perceptions, emotions, behavior, and expectations of others [41]. If the caregiver can appropriately respond to a child's signals, for example, crying, the child can in return learn to understand, organize, and regulate its own emotional state [42]. Consequently, if the child consistently experiences reliable, empathic, and responsive caregiving, it will develop a secure attachment pattern and thus a healthy coping strategy in stressful situations. But what happens if such a “secure base” can only partially be provided? Empirical research in infants, primarily led by the Canadian psychologist Mary Ainsworth, found three attachment patterns: secure attachment, avoidant attachment, and ambivalent attachment [43, 44], and a fourth, later discovered disorganized attachment pattern [45]. *In nuce*, all four patterns vary in a behavioral continuum of exploration, avoidance, intimacy, and clinging to a secure base [13]. For example, infants with a *secure attachment pattern* tend to have a healthy balance between intimacy and independence, being free to explore their environment and at the same time using their parent as a secure base when feeling stressed. Infants with an *avoidant attachment pattern* will be very independent in their exploration behavior and avoid their secure base even when feeling stressed. Infants with an *ambivalent attachment pattern* show almost no signs of independency such as exploration behavior when feeling stressed and behave clingy towards their secure base. Finally, infants with a *disorganized attachment pattern* do not fit into one of the mentioned categories and show rather various attachment behavior; they often show odd exploring behavior and seem to feel very ambivalent towards their secure base (see Table 1).

The Strange Situation Protocol is a standardized research tool for assessing these attachment patterns in young children by observing their attachment behavior in two situations: firstly, in a situation of separation from their parent, and secondly, in a situation of reunion. Being highly dependent on parental care, infants usually fear separation from their caregivers. As children grow older, they learn to cope with this stressful situation in ways that depend on their attachment pattern.

Four corresponding patterns of infant attachment behavior have been found in adult relationships and are analogically categorized as, free (autonomous), dismissive (avoidant), enmeshed (preoccupied) and unsresolved (see Table 1, [46]). The operationalization of the adult attachment interview (AAI) allows the classification of a person into the four main categories of adult attachment patterns. The adult attachment projective picture system (AAP) is another commonly operationalized assessment tool for attachment patterns. Since it is less time consuming and complex, but nonetheless a reliable instrument, it is often used in clinical practice. The clinical implication of the AAP will be discussed later. Both instruments activate a person's early attachment experiences and help to concretize the specific attachment pattern that developed out of the relationship to the primary caregiver. AAI and AAP differentiate between four adult attachment patterns [47].

1. Persons with an *autonomous attachment* representation usually have a coherent narration and are characterized by the capacity to reflect on positive and negative experiences with their caregivers. These individuals are able to show flexible integration and to value their experiences.
2. Individuals with *dismissive attachment* representations are characterized by the deactivation of attachment-related aspects and by certain gaps in their narration that are usually caused by an inability to recall specific childhood memories. They often tend to idealize their parents without clarifying reasons for that idealization. Instead, they tend to make remarks (openly or hidden) that they have been rejected by their parents but claim not to have missed any parental support or felt any negative emotions towards that lack of interest.
3. Individuals with an *enmeshed attachment* representation tend to be emphatic with respect to attachment experiences. They tend to talk a lot about their childhood difficulties, often coming back to that point during the interview, and usually showing that they have not yet come to terms with their childhood problems. Their parents often tended to spoil them, were very anxious, and/or caused guilt feelings when the child tried to be independent and explore its environment.
4. Persons with an *unresolved attachment* representation are characterized by an incoherent narrative style when talking about traumatic experiences [47], with lapses of both reasoning and monitoring.

What all four adult attachment patterns have in common is that the parent's ability to balance intimacy and autonomy, support and independence, and separation/loss and togetherness has a major influence on their children's ability to cope with stress. Furthermore, a well-functioning attachment behavior system is crucial for maintaining emotional stability [48]. According to Attachment Theory, the main cause for the development of disorders is the fear of abandonment and the feeling of vulnerability when the primary caregiver is not available physically or emotionally [47, 49]. Usually, the “inner working models” of attachment patterns will be formed during the first year of life and become more and more stable during the following years [50]. While those patterns remain mostly rather invariant, they still can be influenced by a stable and healthy relationship towards a significant other such as a partner or a psychotherapist [51].

### 3.3. Attachment Behavior and the Palliative Situation

Patients come to Palliative Care when suffering from an incurable, life-threatening disease. It is less about saving the person's life than it is about helping and supporting them as much as possible until their death. Confronted with their own ineluctable death, the patient finds him- or herself in an extraordinarily stressful situation. As described earlier, according to the acquired attachment pattern, different related behaviors will be activated in situations of stress or of a perceived or real threat. The patient's awareness about their increasingly worsening medical condition may trigger threatening feelings of vulnerability. The physical deterioration may result in an increased need for others and thus may confront the patient with fear of dependency [13]. Attachment patterns play a crucial role in the way a patient copes with the situation of approaching death, manifested, for example, in health seeking behavior or the ability to accept and permit needed support from family members and health care providers [52]. The understanding of what lies behind a patient's behavior, wishes, and needs may lead to increased empathetic responsiveness from caregivers and thus contribute to an improvement in the patient's quality of life in Palliative Care. For example, patients having an enmeshed attachment pattern could have their emotional equilibrium maintained by very stable and predictable support [13]. Clinicians knowing and considering the different attachment patterns could both better understand and be less distracted by sometimes contradictory signals and behavior from patients with an insecure attachment pattern and could therefore employ a sufficiently elaborate care approach. Studies have shown that an appreciation of a patient's attachment pattern has beneficial influence on the patient-physician relationship and improves help care outcomes [53, 54]. Moreover, it has been shown, for example, that patients with a secure attachment pattern demonstrate significantly more successful diabetes treatment adherence than those with an insecure attachment pattern (dismissive, enmeshed, or unresolved). However, this effect could partly be attenuated by better patient-physician communication [13, 54, 55]. Results of a study conducted by Milberg et al. [3] indicate that the mediation of security to terminally ill patients (e.g., by providing a familiar environment or fostering trust in a caregiver) induced feelings of control, inner peace, and hope and therefore facilitated patients in dealing with their final parting. Thus, it can be supposed that providing a stable and secure relationship for terminally ill patients can help them to cope better with their condition. This could be explained by the fact that a feeling of security and reliability, the same as with children, can support an adult's cognitive and affective exploration behavior to address adequately existential questions which frequently arise at the end of life [56]. Furthermore, considering a patient's attachment pattern could also help the clinician to influence the success of the therapeutic relationship and thus increase the clinician's ability to grasp the patient's individual needs and wishes in order to support them for the final separation in the best way possible.

Some papers contain case reports describing the different attachment patterns in clinical situations [2, 13]. These case reports give an idea on how individuals with different attachment representations behave when they are in palliative care. The various patient reactions are specified in the following sections in order to elucidate a better understanding of a patient's needs and challenges. 

#### 3.3.1. Secure Attachment Pattern in Palliative Care

Palliative patients with a secure attachment pattern can usually talk openly about positive as well as negative aspects of their life, for example, their relationship to their parents. Memories are easily accessible and emotions are adequately expressed. They openly mourn their situation and express such feelings as fear, anxiety, or anger, which facilitates emotional relief and thus their working through the grieving process. Often, it is sufficient to accompany those patients merely by providing basic support, for example, by helping them in structuring their remaining time or by giving them spiritual support [2]. 

#### 3.3.2. Dismissive Attachment Pattern in Palliative Care

Contact with patients with a dismissive attachment pattern is usually connoted by an impersonal atmosphere and relationship. Emotions are hardly ever spoken about openly; no sadness, no anger, no anxiety is verbally expressed. Their memories about childhood are usually fragmented and the relationship to their parents is idealized without plausible reason. Instead, closer questioning reveals a lack of interest and support on the part of the parents. Such patients are likely to deal with their illness exclusively on a cognitive level, when not completely denying its consequences. Contradictory signals can be given, for example, on the one hand seeking help frequently (e.g., from nurses), while on the other claiming independence and rejecting the help offered. A therapeutic intervention should focus on suppressed feelings, for example, by mirroring latent emotions. Also, involving the patient in active participation in the treatment, for example, could help them to counter threatening feelings of vulnerability and dependency [2, 13]. 

#### 3.3.3. Enmeshed Attachment Pattern in Palliative Care

Patients with an enmeshed attachment pattern often report their childhood memories in a very expansive and tiring way. Their emotional statements seem exaggerated and exalted. They usually had a very dependent relationship with their parents and are likely to maintain this scheme towards other close relatives, for example, the spouse or their own children. Accompaniment of those patients is often time consuming and stressful. The illness is accompanied by extreme senses of anxiety and worry. If emotions are expressed, they occur in the context of long and exhausting sessions. Patients with an enmeshed attachment pattern are usually very demanding, which is apparent, for example, in their high demands for therapy or their need for reassurance to the point that staff can feel overwhelmed. An appropriate intervention could consist of introducing a very structured therapy plan. Emotional predictability can help the patients to establish a secure attachment base and thus experience consistency and reliability [2, 13]. 

#### 3.3.4. Unresolved Attachment Pattern in Palliative Care

It is often hard to construct a clear picture of events from the narrative of an unresolved attached person. Speech is incoherent, interrupted by burdening thoughts and unfinished sentences. Childhood traumas surface frequently in these patients' biographies and have usually never been worked through. Panic attacks, extreme anxiety, or aggressive outbursts are not unusual for them. Conversations about their emotions or feelings about their illness can be held, but information remains confusing and unclear. Often, patients with an unresolved attachment pattern have little or no family and few or no friends to rely on, and if so, relationships are very unstable and complicated. A secure bond should be established to these patients, for example, reducing their mistrust by accompanying them steadily throughout the entire process. Sensible, reliable, and attentive care can help to ease anxiety and panic attacks [2]. 

## 4. Spirituality in Palliative Care

### 4.1. Why Spirituality Is Relevant to Palliative Care

Being torn from everyday life and facing an illness that is incurable and life threatening, it seems natural to search for a deeper reason for one's suffering. It is a human need to search for meaning and purpose, especially when confronted with a disruption of such existential importance, one's own death. Establishing a sense of coherence in exigencies strengthens feelings of self-efficacy and control, thus providing the feeling of safety [57, 58]. Questions, needs, challenges, and distresses arising from the palliative situation address the spiritual dimension of human personality. When facing the word “spirituality,” many physicians respond with a certain kind of restraint because of the word's usual religious implications. In a comprehensive understanding, however, spirituality does not necessarily refer to religious tradition and doctrine but to a fundamental search for transcendence [59]. This means that, in the palliative context, questions such as “Where am I going when death approaches?”, “What will remain when I am gone?”, “Was my life fulfilling?”, “What am I to do as long as I am still able to?”, or “Why me?” may frequently arise. Those questions can bring people into distress as well as be a resource for coping with the nearing end of life.

### 4.2. Spiritual Themes in Palliative Care

For the purpose of this paper, it is crucial to know the main spiritual themes patients are concerned with in order to see how these themes could be reflecting the main points of Attachment Theory. In an international metastudy on the understanding of spirituality among palliative patients, Edwards et al. [60] found seven main themes that constitute a patient's concept of spirituality. It was related to (1) “stories about the whole of life, giving thanks for life” and reflecting the central issues of their life; (2) “relationship with self” in the sense of “self-acceptance” and “self-reconciliation;” (3) “relationships with others” such as family, friends, and caregivers; (4) “relationship with nature and music;” (5) “relationship with God or a higher being” who is able to protect patients “from the fear of death and loneliness;” (6) “hope, meaning, and purpose in life,” whereby themes such as continuation through future generations or the possibility of an afterlife are relevant; and (7) “religious beliefs” in the traditional sense [60]. In the context of this paper, it is worth noting that despite the fact that in the research literature spirituality is often characterized as some kind of intellectual “endowing with meaning,” for the patients as well as the palliative staff members, spirituality is essentially grounded in relationships [60–63]. It is mostly not about finding monadic meaning but meaning within relationships. For example, a common spiritual concept is the meaning of family relationships. Patients see themselves as part of a broader context, the “chain of life” that connects them with previous and coming generations, giving them a feeling of coherence and belonging to something that transcends their passing life [64]. Thus, they are actually meeting the social concept of religion on a fundamental level in the same way that religion in traditional societies is constituted as a ritual community guaranteeing stable relationships and transgenerational cohesion [65]. The term “religion” itself implies relationships, since it is derived from the Latin word *religio* meaning rebond, relation, and attachment. But spirituality in the palliative context goes beyond that social function. It also refers to a feeling of being attached to nature's “circle of life,” permitting reflection about the stories and central issues of their life and their relationship to self. 

### 4.3. Spiritual Needs and Distresses

According to a study by Milberg et al. [3], palliative patients and their relatives consider the feeling of safety provided by a trusted environment or healthcare staff members as the most important factor for feelings of control, inner peace, and hope. To be facing death and therefore the totally unknown means to palliative patients an exposure to uncertainty that can easily touch the shores of fear. Hence, the feeling of safety surely is one of the main issues of a patient's psychological, psychosocial and spiritual needs. In recent times, there have been some approaches to a differentiated conceptualization and measurement of patients' spiritual needs [66, 67], although those are characterized by large heterogeneity [68]. One of the most elaborate instruments, the “spiritual needs questionnaire”, categorizes patients' spiritual needs into five factors: (1) *religious needs/praying*, (2) *existential needs *(*reflection/meaning*), (3) *search attention/connection/relief, *(4)* search for inner peace and *(5)* actively connecting/giving *[69, 70]. This mainly coincides with the pattern of spiritual needs that was identified by the meta-analytical view of Edwards et al. [60] that contained three classes of spiritual needs: (1) the need to “finish business” in the sense of feeling ready to depart without regrets, to forgive, and to be forgiven, (2) the need for “involvement and control” regarding an active preparation for death, and (3) the need for “positive outlook” [60]. We should note that patients' spiritual needs imply two aspects with regard to relationships: on the one hand, a component of activity, of connecting, controlling, being involved, or seeking relief in interaction with others; on the other hand, a component of completion, introspection, seeking to “finish business,” and to come to a calm and peaceful state of mind.

The spiritual distresses pointed out by Edwards et al. [60] directly refer to essential spiritual needs. The main aspect of spiritual distress, an overwhelming fear of death or feeling of uncertainty, refers to the feeling of safety that is evident throughout all spiritual themes. The other essential aspects of spiritual distress are feelings of loss that can concern the self, relationships, or the loss of meaning [60]. The experience of loss concerns relationships (to oneself or other persons, things, etc.) that cannot be given up because they are perceived as essential and indispensable and thus refers in a negative way to the aspect of completion, “finishing business,” and finding peace. Feelings of hopelessness, helplessness, despair, or depression that are additionally related to spiritual distress refer negatively to the aspect of activity: losing control, confidence, or the ability of normal involvement. Feelings of anger or bitterness affect the sense of inner peace; feelings of punishment or judgment by God can be an expression of negative religious coping ([71], for a therapeutical intervention for spiritual pain; e.g., see: [72]). Religious needs, as a part of spiritual needs, however, contain the aspect of introspection (e.g., praying) as well as the aspect of activity (e.g., participation in a religious ceremony). Turning to a higher presence, such as God, is in a way an active attempt at connection and building up relationships, and is at the same time an introspection, thus serving both spiritual needs [73, 74]. 

### 4.4. Transcendence in the Palliative Context

Edwards et al. [60] found “spirituality” and “spiritual care” somehow being used synonymously by patients and healthcare providers, because both were joining in the concept of “caring for relationships.” Notwithstanding the fact that any satisfactory relationship can be supportive in Palliative Care, it is important not to dilute the concept of spirituality into an all-embracing term for “relationship.” In a narrower sense, spirituality always has something to do with transcendence [75], the term transcendence thereby being taken literally and not implying a metaphysical entity, as the example of the “circle of life” demonstrates. Transcendence means in a common sense any idea, expectation, hope, or fear that goes beyond the very physical existence of an individual (for the different meanings of transcendence see [76]). However, to write one's last will and testament or to prepare one's own funeral means to care for something that lies beyond one's own existence and thus can be a source of spiritual reflection. The aspect of transcendence is important to note and not only a matter of dealing with terms, because it relates directly to the situation a palliative patient is confronted with: in the end, he or she will be forced to release the bonds to related persons. Thus, as pointed out before, to “finish business” and to find inner peace are important aspects of the patient's spiritual needs. Spirituality in the palliative situation is not only about maintaining a relationship to loved ones but releasing from them at the same time. The task for relatives, friends, and caregivers is to provide company in the last stages of the patient's life, but in the end every patient has to transcend the final stage unaccompanied, alone. Thus, spirituality in palliative context is a matter of “continuing bonds” [77, 78] while simultaneously coping with separation, a concern regarding how to retain relationships that will inevitably cease. 

### 4.5. “God” in the Palliative Context

Transcendence, however, does not necessarily imply a relationship to a metaphysical entity, to God or a higher being, but of course, it can. Asgeirsdottir et al. [64] report the finding that patients with a nontraditional ecclesiastical spirituality connect transcendent ideas mainly with the larger context of family relationships, whereas patients with a traditional ecclesiastical spirituality tend to center their transcendent relationship on God. The spiritual relationship to God can influence decision making in Palliative Care [79] and affect coping strategies [80] or be a protective factor in preventing disorders [81], whereas van Laarhoven et al. [82] have shown that an image of a nonpersonal higher being can be as important a source of spiritual coping as a personal one. If God in the patient's view is not so much an abstract principle, but someone who personally relates to people, the spiritual relationship in the palliative context can be established in a personal way, too. God can become a personal addressee of the patients hopes, sorrows, and complaints [83], and thus the patient's spirituality can initiate an “internal dialog” of prayer and the verbalization of needs and distresses. It is a frequent occurrence that palliative patients find themselves unable to express all of their fears, spiritual pains, or “unrealistic” hopes, because they feel obligated to treat their relatives with special care and not to intensify their grief. Some palliative patients may feel shame, or even guilt, at perceiving themselves as a long-lasting source of suffering, grief, and trouble to their related persons and thus do not want to cause further problems by not dying in “the right way” [84]. Palliative Care must never lose sight of social distress and pressure, however, which of course are always a dimension of human relationships. From the patient's perspective, the spiritual relationship to God can compensate unexpressed needs as well as be a protected, perhaps secret area for exploring one's own spiritual ideas. Thus, in the patient's view God can take the role of a “transcendent caregiver” and “relative” who can accept the patient's distress. The spiritual relationship to God has been addressed in the research literature from an Attachment Theory perspective by the concept of “Attachment to God” [33, 34], whereas research on spirituality in Palliative Care has yet to be adopted as an area of inquiry in its own right. Granqvist et al. [38] found the spiritual relationship to God to be an important influential factor on the individual attachment working models and attachment patterns. In this way, the spiritual relationship to God can be a corresponding expression of the experiences an individual has had with their caregivers: God is perceived as punitive and distant or nurturing and close when the relationship to the parents or related persons was experienced accordingly [35–37]. While being discussed more controversially, the relationship to God can also compensate for negative attachment experiences [38]. In whatever way formed, a spiritual representation of God as being close and nurturing can provide a secure base in the palliative context and thus be a transcendent companion when death approaches, whereas a punishing or fearful representation could be a source of spiritual distress and threat. 

In the next section, we will discuss how spiritual relationships can be conceptualized in the light of Attachment Theory and which implications this concept entails for Palliative Care.

## 5. Attachment Theory and Spirituality in Palliative Care: Analogies

### 5.1. Theoretical Implications

What do the concepts of Spirituality and Attachment Theory have in common? In our view, one central issue arises in both concepts: establishing a dialectical balance between security and separation. According to the responsiveness of the primary caregiver (and later, of other close persons), different inner working models or “mental representations” of attachment are built during the first year of life and eventually modeled over the lifetime according to later experiences [85]. Bringing someone back on a mental basis by representing a physically absent person requires a high faculty for abstraction and is a developmental task during the early years of life [86, 87]. Hence, for infants, the caregiver has to be physically present, for older children and adults a caregiver can also refer to a mental representation, for example, a transcendent figure. If an individual succeeds in establishing and modeling mental representations, he or she obtains a secure base which plays a crucial role in self-regulation [42]. On the one hand, the developmental task consists of establishing a healthy exploration behavior to foster an open approach to the environment; on the other hand, a healthy attachment system helps to activate resources for maintaining or regaining a stable emotional state in the face of threatening or stressful situations. Following the ideas of Bowlby, exploration behavior and attachment behavior are antagonistic opponents. Individuals can only explore their environment when their attachment system is deactivated. Vice versa, exploration behavior is deactivated when attachment behavior is activated, that is, when the person actively seeks the protection of a caregiver. From the perspective of a child's development, these antagonistic behavior systems are dialectically mediated: infants can only explore their environment and thus overcome the symbiosis with their primary caregiver(s), when at the same time they feel they can rely on a secure base. 

**Figure 1:**
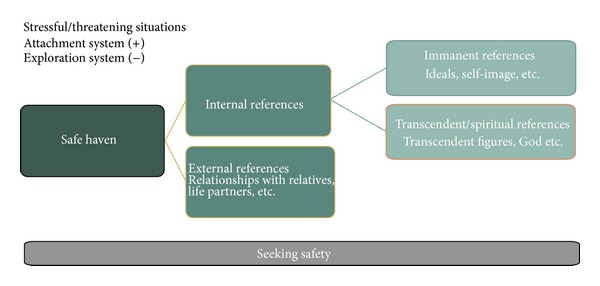
Seeking for safety in stressful/threatening situations.

Two different terms are used in Attachment Theory to describe the feeling of security in the attachment system. Attachment behavior is activated in a threating or stressful situation, while at the same time exploration behavior is deactivated. The individual seeks safety and is not interested in any kind of exploration. In the context of these situations, resources for reassurance and calming are called a “safe haven”. According to attachment theory, this can only be provided by primary caregivers such as the parents. The term “haven” denotes that the individual takes a “step back” towards a caregiving attachment figure (enters the port) or, returning to the term explained earlier, engages in a “religio” (rebond, relation, attachment). In this paper, we assume with regard to our discussion of the concept of spirituality that a “safe haven” can be provided by an external reference, that is, a real person, as well as by an internal reference (see Figure 1). The internal reference itself can refer to two different types: an immanent internal reference, for example, representations of ideals, self-image, mother or father image; and a transcendent/spiritual internal reference, for example, transcendent figures such as God. These images provide a possible consolation and feeling of security for an individual in a catastrophic situation, and thus the stabilizing function of a transcendent figure need not depend on its real presence. A symbolic reference, for example, a cross, or its mere representation could be enough. 

**Figure 2:**
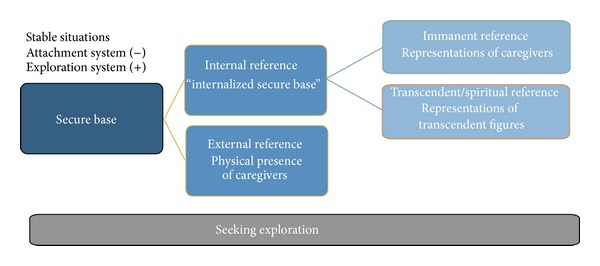
Seeking for exploration in stable situations.

The second aspect of security relates to situations in which there is no perceived threat, or in which consolation had been successful; thus, attachment behavior is deactivated while exploration behavior is activated. In such situations, exploration is possible if individuals can relate to a “secure base” from which the environment can be explored. As shown in Figure 2, a “secure base” can be provided by an external as well as an internal reference. The internal reference represents an internalized secure base, the “inner working model,” which is built in the course of an individual's development and that serves as a regulating resource whenever the caregiver is not present. This is made possible by a mental representation of the caregiver that has a stabilizing and supporting influence, even when that caregiver is not physically present. The internal reference is in turn divided into immanent and transcendent references of representations. The caregiver function that had provided a feeling of security has now changed: while before in the situation of the “safe haven,” the caregiver was the goal of the activated attachment behavior, the caregiver now represents the starting point for exploration behavior. 

**Figure 3:**
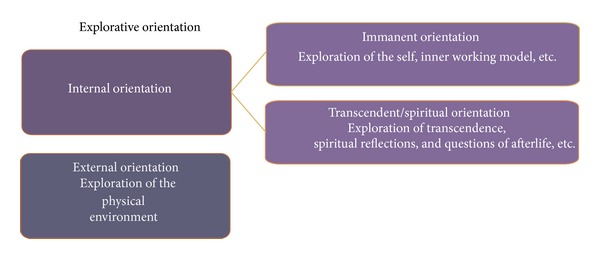
Explorative orientation.

This starting point allows different modes of exploration, as shown in Figure 3. The external orientation refers to the exploration of the physical environment, other people, and so forth. The internal orientation—which surely is more relevant for the palliative situation—can again be divided into an immanent and a transcendent or spiritual orientation. The immanent orientation implies exploration of the self, inner thoughts, ideas, questions of meaning, and so forth. Transcendent/spiritual orientation concerns questions of afterlife, spiritual meaning of life, religious questions, and so forth.

We now can localize spirituality in the theoretical framework of Attachment Theory: spirituality, as a simultaneous search for a secure relationship as well as transcendence, can be conceptualized as a variant of attachment behavior. Spiritual representations can serve as an aspect of security in threatening situations, whereas the spiritual search for transcendence can be conceptualized as a part of exploration behavior. Thus, spirituality plays a crucial role in the dialectic of security and exploration. But what does it mean for Palliative Care? 

In our view, the aspect of spirituality as a certain form of attachment behavior pertains directly to the demands and special qualities of the situation that a terminally ill patient is confronted with.

As mentioned earlier, the palliative situation is an extremely threatening one in which attachment behavior is activated. The patient's spirituality can serve as a source of consolation and reorganization and can help them to regain strength and emotional stability. In the clinical situation, it is thus important for caregivers to be able to recognize a person's current state within the attachment system. If an individual's attachment behavior is activated, he or she will likely—in a manner depending on their specific attachment pattern—seek a “safe haven” in which they can deactivate their attachment behavior. Spirituality seems to be an important resource for this task. It can be hypothesized that patients in this situation will be less likely to explore or engage in spiritual reflection about new and perhaps unknown aspects of their spirituality; they rather tend, we assume, to relate to the better known aspects of their spirituality. Thus, it can be important to know a patient's spiritual orientation in order to support a better mediation between external and internal representations. Religious symbols, prayers, conversations, and so forth can support this mediation and strengthen the consoling function of spiritual representations. 

Perhaps less obvious is the aspect of exploration in Palliative Care: patients in the palliative context are confronted with the totally unknown aspect of their own death, an experience that transcends the realm of their known relations. This “unknown” must not only be a source of anxiety and insecurity, forcing the patient into a rear-guard action, but it can also be a challenge for exploration. The confrontation with one's own death is thereby not so much a question of social exploration or orientation in the physical environment. Death as the ultimate unknown and mystery affects the domain of transcendence and therefore of spirituality. Thus, it is not surprising that the necessity to reflect on spiritual themes takes on greater significance when one is confronted with a life-threatening illness and the possibility of one's own dying [88, 89]. Spiritual reflection should not aim for empty promises or placation, but it seems to be more the adequate answer to the peculiarity of the situation that palliative patients find themselves in. As will be shown in the following examples, health caregivers should not only be able to recognize but also support situations in which the attachment system is stabilized and the exploration behavior is activated. A person's spiritual reflections do not necessarily indicate a need only for security, but it may also indicate a sense of curiosity and an engagement in exploration of the unknown. 

In the palliative context, spirituality refers to both aspects of attachment behavior: the aspect of security as well as the aspect of exploration. As described earlier, exploration in the spiritual dimension means interaction with transcendence. This interaction with transcendence can occur on an intramundane level, for example, in drawing up the will or in seeking a meaningful position in the “chain of life.” It can also take place on an extramundane level and, for example, contain representations of life after death, or the beyond, and so forth. (of course the opposite of these representations can be possible as well, by rejecting the acceptance of such representations). Successful exploration behavior in the palliative situation does not mean having a clear and polished position toward transcendent questions but rather implies an anxiety-free openness toward those questions—by whatever means they are answered or not. A secure base in relationships that are perceived as being steady and protective forms the foundation of transcendent exploration. There is no doubt about the crucial role of relatives, friends, and caregivers who can mediate the patient a feeling of security and comfort and thereby activate and support the resources the patients need to confront their own death. Equally important, though, are the internal references—like having a stable relationship to oneself or to transcendent figures—from which the patients can experience feelings of security and boundedness according to their attachment pattern. In the context of the palliative situation, the relationship to transcendent figures gains a particular relevance. It would be insufficient to conceptualize the spiritual relationship to transcendent figures only as a mental representation of human relationships [90]. For a patient, the spiritual relationship would then only be a pale reflection of perceptible and tangible interactions. But the representation of transcendent figures, for example, God, concerns the aspect of transcendent exploration as well as God itself implies the unknown and uncertain. So to speak, God is “resident in two worlds,” being perhaps the most intimate confidant and a totally unknown one at the same time. In the palliative situation, this makes the position of the spiritual relationship to transcendent figures exceptional, figures who can be intermediaries between the secure and the unknown, between continuation and transcendence. Without any doubt, the spiritual relationship and transcendent exploration can also be sources of anxiety and distress. Palliative caregivers have to keep a patient's attachment pattern in focus in order to understand in what ways it may influence the patient's manner of structuring attachment and exploration behavior.

### 5.2. Practical Implications

The concept can help to identify the patient's attachment signals and to provide an adequate response. Palliative treatment, in considering Attachment Theory and Spirituality, has to take into account two aspects of attachment behavior: initially, it must recognize the fundamental situation of the patient. Is the patient in a situation of threat and thus is his or her attachment pattern highly activated? In this case, it is important to establish a safe atmosphere and to provide a stable presence in order to reassure the patient and restabilize their attachment behavior. Or is the patient in a situation of stability that gives him or her the possibility to engage in exploration, for example, spiritual reflection? In that case, it would be important to provide a secure base, but not to disturb the patient's exploration—due to the fact that spiritual reflection does not necessarily imply thoughts of consolation, but can have dark and unsatisfying implications. The task is to transform fear into security, in order to make the activation of the exploration system possible. In this way, the patient may find resources in their internal immanent or transcendent representations that will help them to cope with situations of fear. The other aspect concerns the different attachment patterns. Palliative Care, as we described earlier, must take into account the different ways in which a person organizes relationships and expresses needs. We now offer some examples from clinical praxis in order to illustrate how the concept can help to understand patient needs. 

Earlier, we said that the patient's own spirituality could be a source of anxiety. This can be shown by the case study of a patient who suddenly started having panic attacks a few days before his death [91]. He reported that he was frightened and that nothing in his life had been of any value. He continued that nothing he created would remain, that he did not believe in God or any other higher power, but rather that life would just end; there would only be darkness and emptiness. This thought frightened him again, so that finally he had to be given a strong sedative which continued to be administered until his death [91]. In our concept, the patient found himself in a fearful and threatening situation that highly activated his attachment system. His fears were about the darkness and emptiness which would overcome him upon death, as well as the senselessness of his life, and thus affected the aspect of transcendence in a negative way. In this situation of fear, the patient longed for a safe haven, but he could not rely on a secure immanent or spiritual representation that would have given him consolation. He was not indifferent to his immanent or transcendent afterlife, but he was not able to explore this aspect without fear: the absence of a safe haven means the absence of a secure base for exploration, as well. Unfortunately, the patient's physical condition made it impossible for him to engage in long conversations about his spirituality. Perhaps, it would have been helpful to have talked with him about the value and the meaning of his life while he was still capable of doing so. Also, a gentle exploration with him, together with another person, about what might happen upon his death might have helped this patient not to wait with this reflection until just before his death; the person could have asked him what his belief of “darkness” meant to him personally, since that belief obviously frightened him so much. Perhaps he would have found a safe haven and, later on, a secure base for fearless exploration of his spiritual thoughts. Possibly, this patient seemed self-reliant due to his attachment style and did not ask for help until he could not hold back due to his panic attacks. 

Petersen and Koehler [2] report a case of a 75-year-old woman with a secure attachment pattern. She was brought to the palliative unit with a liver carcinoma. In the beginning, she felt strong physical pain but was able to describe it precisely so that the doctor could help her effectively. During the daily conversations with the doctor, the patient talked openly about her life and valued negative as well as positive experiences. She had felt that in her childhood “both of her parents were available for her all the time” [2]. She experienced rough times during the war and in the following years but was able to build a new and satisfying life. She was happily married until her husband's death some years ago and had established a warm and close relationship to her son and his family. All throughout her stay in the palliative unit, she was surrounded by her family and close friends, who provided a secure base for her exploration of the self and of questions of transcendence. Daily conversation helped her to reflect on her life and to value the way she had lived it, including good as well as bad episodes. She could talk openly about her anxiety to die and was able to mourn. The conversations about her life, her illness, and her death, including all the emotions that were experienced in the process, helped her to accept her situation. Before her death, she asked for spiritual support from a priest. Finally, the patient was able to let go and died reconciled and in a state of calmness. 

In contrast, persons with a dismissive attachment pattern may seem indifferent to their illness, but in fact they feel fear and anger that they do not know how to cope with. Transcendent exploration may be performed in an impersonal and distanced way, indicating no personal or intrinsic interest. Nevertheless, it is important to recognize the emotions of fear and forsakenness hiding behind the cover of distance and to encourage and support exploration behavior sensitively. A case study of a person with dismissive attachment behavior is reported by Tan et al [13]. A 47-year-old woman was admitted to the palliative-care unit with ovarian cancer. The patient had been self-reliant and had avoided close relationships throughout her life. Instead, she was very devoted to her work. Her fear of dependence was apparent in her ambivalent behavior: on the one hand demanding help and more support from the staff, on the other hand rejecting treatment when it was offered. Those contradictory signals became not only a challenge for the staff, but also made the patient suffer more than necessary, as well. Reluctantly, she agreed to accept individual counseling. It turned out that she wished to die and did not see any point in her continued living; at the same time, her spiritual beliefs prevented her from ending her life deliberately. Based on the understanding of her dismissive attachment pattern, health care providers were instructed to ensure the patient's participation in treatment as much as possible. In terms of spiritual needs, the counseling sessions helped her to structure her spiritual exploration. She began to communicate her feelings—which led to less contradictory signals—and to accept and better value human relationships. Instead of dying alone, as she had wished to at the beginning, she was able to accept support from her mother and her friends, who were beside her when she died [13]. 

Persons with an enmeshed attachment pattern may not exhibit any exploration behavior at all, because confrontation with their own death activates strong feelings of fear and panic. Therefore, constantly animating them to explore may trigger anxiety. Possibly, a careful and sensitive approach is indicated that takes the patient step-by-step toward a tolerable level of exploration. Tan et al. [13], for example, describe a 55-year-old woman with terminal cancer. She showed strong feelings of anxiety toward her death, concomitant with significant panic attacks. The patient reported that while in her childhood her physical needs had always been satisfied, she had experienced a lack of emotional support. She always had been insecure, despite the success in her career and loving friends and husband. At the slightest change of her symptoms, the patient saw herself confronted with catastrophic thoughts and intense fears of death. The frequent attempts at reassurance and intensive attention seemed to calm the patient's fears for a short time, but to the point that some caregivers felt overwhelmed by the patient. Based on the knowledge of the patient's attachment pattern, a structured plan was elaborated which had to be strictly followed. The structure helped the patient find a certain regularity in her treatment, which enabled her to control her fears better. Regular meetings with family, physicians, and psychiatrists helped her to express her anxiety to die [13]. 

Patients with an unresolved attachment pattern expectedly show very varied and ambiguous exploration behavior. Besides continuous and sensitive spiritual support, it is important not to be misled by the patient's alternating and possibly aggressive behavior, but instead to support the patient in structuring their transcendent exploration. Petersen and Koehler [2] give the example of a 68-year-old woman who was admitted to the palliative care unit with untreated breast cancer. The patient was very aggressive and defensive, allowing little to no treatment in the beginning. After a few days, and consistent but gentle approaches towards her, she agreed to show her open breast wound and allowed it to be bandaged. Step by step, the patient began to develop some trust toward her caregivers. When asked questions about herself and her life, she often fell into a confused or aggressive state of mind; sometimes, her answers were clear and structured, but no more words than necessary came over her lips. Her childhood memories were fragmented; she often did not even finish her sentences when asked about it, but rather fell into a trance-like state. Nevertheless, it became clear that she had been maltreated when she was little, and that in this short amount of time it would be impossible to provide sufficient therapy to ease her anxieties. Her extreme anxiety led to emotional outbursts; sometimes she cried, and sometimes she became highly aggressive towards staff members. Her caregivers tried to provide the patient with a sense of security through reliable and time-intensive care. A few days before her death, the patient fell into a serious delirium that could be treated successfully. 

Although it is obviously helpful to know a patient's attachment pattern and spiritual orientation, there are difficulties in their assessment in the palliative context. When patients enter the palliative care unit, they usually get admitted when their health condition is declining, and they often stay only for a couple of days, if their health condition gets better again. An AAI may be a possible evaluation instrument, but it is very time consuming and complex and can be very exhausting for the patient. The AAP, on the contrary, seems less intrusive and also takes a shorter time to evaluate, although reliability is just as good as the AAI. Therefore, it seems to be a more accurate instrument in the context of palliative care. First trials to implement the AAP in the palliative-care context have been made by the working group of Hloucal et al. [92]. Although an important element, the spiritual dimension of attachment is a problematic aspect in the evaluation of the AAP. Many spiritual themes in the AAP (which fall into a certain pattern of evaluation) are classified as being “spooky,” and thus indicate traumatic experiences. This could be a serious problem, since first results seem to indicate that palliative-care patients often return to the spiritual dimension during the interview as a secure or insecure base of attachment. A patient will thus easily be classified as “disorganized,” even when his or her spiritual resources may be helpful and a source of strength [91]. These findings confirm the importance of the spiritual dimension of attachment: if this dimension is not integrated in the concept of attachment, it could lead to wrong assumptions about one person's attachment pattern and thus misjudge an important resource of coping with the own death. Further research has to show how Attachment Theory and spirituality can be integrated not only on a theoretical base but in terms of measurement as well. 

## 6. Conclusion

The aim of this paper was to discuss and explore the interrelation between two concepts which are important to Palliative Care, namely, Attachment Theory and the concept of spirituality. We found that both concepts have two aspects in common: the aspect of security and the aspect of exploration. More precisely, spiritual relationships can be considered analogous to the concept of “safe haven” and “secure base” in Attachment Theory, whereas the spiritual search for transcendence as analogous to “exploration behavior.” For the category of security as well as for the category of “exploration,” two ideal-typical subcategories have been, respectively, conceptualized: an external and an internal division. The internal subcategories can in turn refer to an immanent internal and a transcendent/spiritual division. This conceptualization seems important in Palliative Care, because spirituality cannot be seen as a monadic superimposed variable that individuals may or may not refer to, but more as a basic need of all humans constituted as a certain expression of attachment behavior. Spirituality can be an adequate answer of attachment behavior to the peculiarity of the palliative situation. Spiritual seeking as a part of attachment behavior is activated and becomes especially relevant when an individual is confronted with a highly stressful situation, such as a palliative patient, is confronted with. Following the line of reasoning in this paper, this would imply some adjustments in Attachment Theory. For example, in the Adult Attachment Projective Picture System (AAP)—that is a commonly operationalized assessment tool—a person referring to a transcendent internal reference would likely be classified as having an insecure attachment pattern, even when perceiving it as a source of support and strength [91]. As we discussed earlier, positive effects of palliative treatment adequate to attachment have been shown. Knowing how to support and activate the patient's spiritual exploration may enhance this effect and could thus add to the patient's end-of-life quality. In the end, no caregiver, relative, or friend can take the confrontation with transcendence off the patients shoulder. Transcendence will never be answered in a completely satisfying way, but it will always refer to an unknown [93]. Transcendent exploration can only be carried out successfully in an individual way. At the beginning, as well as at the end of life, we face an unknown that frightens us and that we must confront, preferably from a secure base.

## Figures and Tables

**Table 1:** Attachment patterns in children and adults.

	Secure	Insecure		
Children	Secure	Avoidant	Ambivalent	Disorganized
Adults	Autonomous	Dismissive	Enmeshed	Unresolved
